# Impact of immune-related adverse events on treatment outcomes in advanced esophageal squamous cell carcinoma treated with immune checkpoint inhibitors

**DOI:** 10.3389/fimmu.2026.1741482

**Published:** 2026-02-05

**Authors:** Tianhang Zhang, Xiao Chen, Jianhua Wu, Jiasong Li, Zhukun Qin, Ruijie Cao, Wei Guo, Zhanjun Guo, Haiyan Fan

**Affiliations:** 1 Department of Rheumatology and Immunology, The Fourth Hospital of Hebei Medical University, Shijiazhuang, China; 2 Animal Center, The Fourth Hospital of Hebei Medical University, Shijiazhuang, China; 3 Department of Gastroenterology and Hepatology, The Fourth Hospital of Hebei Medical University, Shijiazhuang, China; 4 Laboratory of Pathology, Hebei Cancer Institute, The Fourth Hospital of Hebei Medical University, Shijiazhuang, China

**Keywords:** esophageal squamous cell carcinoma, immune checkpoint inhibitors, immune-related adverse events, prognosis, therapeutic efficacy

## Abstract

**Background:**

While immune-related adverse events (irAEs) are associated with better prognosis in advanced esophageal squamous cell carcinoma (ESCC), the prognostic impact of single-organ irAE (uni-irAE), multiple-organ irAEs (multi-irAEs), and organ-specific irAEs remains poorly understood. This study aimed to address this gap by evaluating the effects of various irAEs on survival and characterizing the co-occurrence patterns of multi-irAEs in ESCC patients.

**Methods:**

We retrospectively analyzed 213 ESCC patients treated with immune checkpoint inhibitor (ICI), dividing them into non-irAE, uni-irAE, and multi-irAEs groups to compare their efficacy and prognosis. Baseline characteristics and efficacy outcomes were compared by Chi-square test. Prognostic analysis was performed using Kaplan-Meier survival analysis with the log-rank test and Cox proportional hazard models. The Mann-Whitney U test was used to compare the time to onset of irAEs. Additionally, logistic regression analysis was conducted to identify risk factors associated with the development of multi-irAEs.

**Results:**

Patients who developed irAEs exhibited a significantly higher disease control rate (DCR) compared to patients without irAEs (94.9% *vs*. 82.1%, *p* = 0.007). This was observed in both the uni-irAE group (93.4% *vs* 82.1%, *p* = 0.036) and as a trend in the multi-irAEs group (100% *vs*. 82.1%, *p* = 0.078) when compared to the non-irAE group. Multivariate analysis revealed that the development of uni-irAE was an independent protective factor for both progression-free survival (PFS; hazard ratio [HR] 0.57, 95% confidence interval [CI] 0.39-0.83, *p* = 0.003) and overall survival (OS; HR 0.64, 95% CI 0.44-0.95, *p* = 0.028). Similarly, multi-irAEs were identified as an independent protective factor for OS (HR 0.41, 95% CI 0.20-0.86, *p* = 0.019). Analysis of co-occurrence patterns showed that endocrine irAEs were frequently leading to multi-irAEs. Furthermore, a multivariate Cox regression confirmed that endocrine irAEs and mild (grade 2 or lower) irAEs were independently associated with favorable survival outcomes.

**Conclusion:**

The occurrence of both uni-irAE and multi-irAEs was associated with favorable prognosis in ESCC patients treated with ICIs. Furthermore, patients who developed endocrine irAEs or mild irAEs also demonstrated improved efficacy, suggesting their potential as clinical response markers for a positive response to therapy. This finding emphasizes the necessity of vigilant monitoring and early intervention for irAEs in patients undergoing ICIs.

## 1 Introduction

Esophageal cancer (EC) is a major malignant tumor of the digestive system, ranking as the eighth most common cancer and the sixth leading cause of cancer-related mortality worldwide, with an estimated 604,000 new cases and 544,000 deaths annually (1, 2). Under the influence of genetic factors and various environmental factors, the incidence and mortality rates of EC exhibit variations across different geographical regions, genders, and ethnicities (3). Particularly remarkable is China, where esophageal squamous cell carcinoma (ESCC) constitutes the most prevalent subtype (approximately 90% of all esophageal cancer cases), and accounts for more than half of the new cases and deaths worldwide (4, 5).

Early-stage EC is primarily treated with endoscopic therapy and surgical resection (6). However, many patients are unsuitable for surgery because of the location in the upper thoracic segment of the esophagus, and many others are initially diagnosed at an advanced stage and thus lose the opportunity for surgery (6). Thereby, the standard systemic treatment, mainly encompassing chemotherapy and immunotherapy, is particularly crucial for unresectable or advanced EC. Prior to 2017, paclitaxel, platinum and fluorouracil were commonly utilized chemotherapeutic agents that dominated in the treatment for a long time, but the five-year survival rate remained below 5%, and even lower for advanced ESCC patients (7, 8). The treatment landscape was fundamentally transformed by the introduction of immune checkpoint inhibitors (ICIs), which target the programmed death 1 (PD-1)/programmed death‐ligand 1 (PD-L1) axis to enhance T-cell-mediated anti-tumor immunity (9). Since 2017, there have been continuous developments in the application of immunotherapy for advanced ESCC. The clinical exploration of immunotherapy began with the ATTRACTION-1 trial of nivolumab and the KEYNOTE-181 trial of pembrolizumab, which focused on its application in second-line and subsequent-line treatments (10–12). Then this success was subsequently extended to the first-line setting, with large-scale Phase III clinical trials (such as KEYNOTE-590 trial of pembrolizumab, CheckMate-648 trial of nivolumab, ORIENT-15 trial of sintilimab, and RATIONALE-306 trial of tislelizumab) demonstrating the significant efficacy of ICIs (13–16). Based on the results of these studies, both the 2023.V3 National Comprehensive Cancer Network (NCCN) Guidelines and the Chinese American Society of Clinical Oncology (ASCO) Guidelines for 2023 recommend the use of various immunotherapy regimens in combination with chemotherapy as first-line or subsequent-line treatment options for ESCC (7, 17, 18). However, the initiation of ICIs can result in the emergence of immune-related adverse events (irAEs), which represent a double-edged sword in cancer immunotherapy. On one hand, irAEs can affect multiple systems and are potentially life-threatening (19–23). On the other hand, their underlying pathophysiological mechanisms, including T-cell-mediated cytotoxicity, elevated autoantibody levels, inflammatory cytokine release, and complement-mediated inflammation, may reflect a robust anti-tumor immune response, thereby contributing to favorable therapeutic outcomes (20, 24). Accumulating evidence indicates that irAEs serve as a biomarker for improved outcomes in various malignancies, including lung cancer, hepatocellular carcinoma, gastric cancer, and renal cell carcinoma (19, 20, 23, 25–34). Research is now moving beyond the binary presence of irAEs to investigate the prognostic impact of single-organ irAE (uni-irAE), multiple-organ irAEs (multi-irAEs), and organ-specific irAE (26–30, 35). While the occurrence of irAEs predicts better outcomes in EC patients, the relationship between subtypes of irAEs (uni-irAE, multi-irAEs, and organ-specific irAE) and outcomes remains particularly underexplored (36–38).

Therefore, we conducted this study to determine how irAE characteristics, such as number, affected organ, and severity, influence therapeutic efficacy and prognosis in ESCC patients undergoing ICI therapy. Furthermore, we investigated the specific irAE co-occurrence patterns across multiple organs.

## 2 Materials and methods

### 2.1 Patient

This retrospective study enrolled 213 patients with advanced ESCC received anti-PD-1 antibodies who were admitted to the Fourth Hospital of Hebei Medical University between August 2019 and April 2025. Inclusion criteria were as follows: a. Pathologically confirmed squamous cell carcinoma, b. Diagnosis of advanced unresectable tumor according to NCCN guidelines (17); c. First-line treatment with chemotherapy combined with immunotherapy, with or without local radiotherapy and targeted therapy, d. Receipt of at least three cycles of ICIs; d. Eastern Cooperative Oncology Group (ECOG) score ≤2; f. Availability of baseline and post-treatment imaging evaluation data, with at least one completed imaging assessment. Exclusion criteria were as follows: a. Preexisting autoimmune diseases; b. Pathological types other than squamous cell carcinoma; c. History of other malignant tumors; d. ECOG score ≥3 or multiple organs failure.

The study systematically collected data including baseline levels of neutrophil-to-lymphocyte ratio (NLR), as well as clinical variables including gender, age, ECOG score, tumor node metastasis (TNM) stage, tumor differentiation, tumor site, PD-L1 expression, history of surgery, chemotherapy regimen and the type of irAEs.

All procedures were conducted in line with the Helsinki Declaration of 1964 and its later amendments and have been reviewed and sanctioned by the Ethics Committee of the Fourth Hospital of Hebei Medical University. Due to the retrospective design of the study, the requirement for informed consent was waived.

### 2.2 Treatment efficiency assessment and follow-up

IrAEs were identified and assessed by physicians or pharmacists, and all events were graded for severity and classified according to the Common Terminology Criteria for Adverse Events (CTCAE) version 5.0 (Common Terminology Criteria for Adverse Events (CTCAE)). In this study, we defined mild irAEs as those of grade 2 or lower while severe irAEs as those of grade 3 or higher. We defined a “uni-irAE” as an irAE affecting a single organ, and “multi-irAEs” as those affecting two or more organs. The affected organs included dermatologic, gastrointestinal, endocrine, cardiac, pulmonary, and hepatic. In the analysis of organ-specific irAEs, the other patients’ group was defined as all patients excluding those with the corresponding organ-specific irAEs. In this study, we performed group comparisons across three distinct criteria: by the number of affected organs (non-irAE, uni-irAE, multi-irAEs), by the organ-specific (organ-specific irAE, other patients), and by the severity (non-irAE, mild-irAE, severe-irAE).

Patients received anti-PD-1 therapy in 21-day cycles. Tumor response was evaluated radiologically using computed tomography (CT) scans or magnetic resonance imaging (MRI) scans every 2 to 3 treatment cycles, with assessments based on the Response Evaluation Criteria in Solid Tumors (RECIST) version 1.1 until disease progression or death occurred (39). Complete response (CR) was defined as the disappearance of all target lesions, and partial response (PR) was defined as at least a 30% decrease in the sum of diameters of target lesions. Progressive disease (PD) was defined as at least a 20% increase in the sum of diameters of target lesions with an absolute increase of at least 5 mm or the appearance of new lesions. Stable disease (SD) was defined as changes in tumor size that did not meet the criteria for PR or PD. The disease control rate (DCR) was the proportion with a CR, PR, or SD.

All patients were monitored through re-hospitalization, outpatient clinic visits, and telephone follow-ups until death or loss of contact for any reason. Survival endpoints included: progression-free survival (PFS), defined as the time from immunotherapy initiation to disease progression, death from any cause, or data censoring at the last follow-up; overall survival (OS), defined as the duration from treatment start to death or censoring at the last follow-up; The end point of follow-up was 10 October 2025 or the date of death.

### 2.3 Data analysis

Statistical analyses were conducted using SPSS version 27.0 and R version 4.5.0. Pearson’s chi-square test and Fisher’s exact test were performed to compare the groups. Survival outcomes were assessed using the Kaplan-Meier survival analysis with the log-rank test. Univariate and multivariate Cox proportional hazard models were used to assess the prognostic impact of irAEs. Univariate logistic regression analyses were employed to evaluate risk factors for the development of multi-irAEs. The time to onset of irAEs was compared between the organ-specific irAE group and the multi-irAEs group using the Mann-Whitney U test. Univariate and multivariate Cox proportional hazard models for OS and PFS were constructed only for organ-specific irAE with more than 10 cases. Two-tailed *p* values were calculated and statistical significance was defined as *p* < 0.05.

## 3 Results

### 3.1 Baseline characteristics of the patients

The study incorporated a total of 213 patients diagnosed with advanced ESCC, all of whom were administered anti-PD-1 antibodies, including sintilimab, tislelizumab, camrelizumab, pembrolizumab, nivolumab, toripalimab, and serplulimab (**Supplementary Table S1**). We compared the incidence of irAEs among different types of ICIs with more than 5 reported cases and found no statistically significant differences (**Supplementary Table S2**). We classified these patients into three groups according to the number of affected organs: 134 (62.91%) with non-irAE, 61 (28.63%) with uni-irAE, and 18 (8.45%) with multi-irAEs. The patients’ baseline characteristics are shown in **Table 1**, no significant difference was observed among the non-irAE group, uni-irAE group and multi-irAEs group in terms of median Age, NLR, age, gender, ECOG score, TNM stage, tumor differentiation, tumor site, PD-L1 expression, history of surgery, chemotherapy regimen.

**Table 1:** Baseline characteristic of the patients.

Variables	Total (n = 213)	Non-irAE (n = 134)	Uni-irAE (n = 61)	Multi-irAE (n = 18)	*P*-value
Median Age (IQR)	68.00 (62.00, 73.00)	68.00 (61.00,72.00)	67.50 (62.00,74.25)	69.50 (67.25,73.25)	0.458
NLR (ratio)	3.46 (2.40, 4.79)	3.48 (2.52,5.03)	3.24 (2.23,4.43)	3.91 (2.79,4.71)	0.485
Gender					0.145
Female	62 (29.11)	36 (26.87)	23 (37.70)	3 (16.67)	
Male	151 (70.89)	98 (73.13)	38 (62.30)	15 (83.33)	
Age Group					0.148
<65	67 (31.46)	44 (32.84)	21 (34.43)	2 (11.11)	
≥65	146 (68.54)	90 (67.16)	40 (65.57)	16 (88.89)	
NLR					0.589
NLR<3	83 (39.15)	50 (37.59)	27 (44.26)	6 (33.33)	
NLR≥3	129 (60.85)	83 (62.41)	34 (55.74)	12 (66.67)	
ECOG					0.499
≤1	175 (82.16)	108 (80.60)	53 (86.89)	14 (77.78)	
>1	38 (17.84)	26 (19.40)	8 (13.11)	4 (22.22)	
TNM stage					0.103
II	22 (10.33)	10 (7.46)	11 (18.03)	1 (5.56)	
III	82 (38.50)	49 (36.57)	26 (42.62)	7 (38.89)	
IV	109 (51.17)	75 (55.97)	24 (39.34)	10 (55.56)	
Tumor differentiation					0.243
Medium to high	180 (84.51)	113 (84.33)	54 (88.52)	13 (72.22)	
Low	33 (15.49)	21 (15.67)	7 (11.48)	5 (27.78)	
Tumor site					0.600
Upper	37 (17.37)	23 (17.16)	11 (18.03)	3 (16.67)	
Middle	69 (32.39)	47 (35.07)	17 (27.87)	5 (27.78)	
Lower	51 (23.94)	35 (26.12)	12 (19.67)	4 (22.22)	
Other	56 (26.29)	29 (21.64)	21 (34.43)	6 (33.33)	
PD-L1 expression					0.618
Negative	73 (34.27)	43 (32.09)	25 (40.98)	5 (27.78)	
Positive	1 (0.47)	1 (0.75)	0 (0.00)	0 (0.00)	
Unknown	139 (65.26)	90 (67.16)	36 (59.02)	13 (72.22)	
History of surgery					0.652
No	28 (13.15)	16 (11.94)	11 (18.03)	1 (5.56)	
Yes	57 (26.76)	35 (26.12)	16 (26.23)	6 (33.33)	
Number of metastases					0.436
≤1	152 (71.70)	92 (68.66)	47 (77.05)	13 (76.47)	
>1	60 (28.30)	42 (31.34)	14 (22.95)	4 (23.53)	
Chemotherapy regimen					0.321
Others	33 (15.49)	18 (13.43)	13 (21.31)	2 (11.11)	
TP	180 (84.51)	116 (86.57)	48 (78.69)	16 (88.89)	

IrAEs, immune-related adverse events; ESCC, esophageal squamous cell carcinoma; Uni-irAE, single-organ irAE; Multi-irAEs, multiple-organ irAEs; NLR, Neutrophil to Lymphocyte Ratio; ECOG, Eastern Cooperative Oncology Group; TNM, tumor-node-metastasis; PD-L1, programmed cell death ligand 1.

### 3.2 Association between irAEs and treatment efficacy

The overall DCR of PD-1 treatment was 86.9% (n=185), with 94.9% (n=75) in the non-irAE group whereas 82.1% (n=110) in the irAEs group. As shown in **Table 2**, the occurrence of irAEs was significantly associated with a higher DCR compared to the non-irAE group (*p* = 0.007). This association remained significant for patients with uni-irAE (*p* = 0.036) and showed a strong trend for those with multi-irAEs (*p* = 0.078). There was no significant difference in DCR between the uni-irAE group and multi-irAEs group. These data demonstrated that the occurrence of uni-irAE was associated with an improved treatment response.

**Table 2:** Response to ICI of irAEs, non-irAE, uni-irAE, and multi-irAEs groups in ESCC patients.

Comparison	PD	SD	PR	CR	DCR	*P-*value
Non-irAE group vs irAEs group						0.007
Non-irAE group	25	81	28	0	82.1%	
irAEs group	3	56	18	2	94.9%	
Non-irAE group vs Uni-irAE group						0.036
Non-irAE group	25	81	28	0	82.1%	
Uni-irAE group	3	42	14	2	93.4%	
Non-irAE group vs Multi-irAEs group						0.078
Non-irAE group	25	81	28	0	82.1%	
Multi-irAEs group	0	14	4	0	100%	
Uni-irAE group vs Multi-irAEs group						0.569
Uni-irAE group	3	42	14	2	93.4%	
Multi-irAEs group	0	14	4	0	100%	

IrAEs, immune-related adverse events; Uni-irAE, single-organ irAE; Multi-irAEs, multiple-organ irAEs; ESCC, esophageal squamous cell carcinoma; SD, stable disease; PR, partial response; PD, progressive disease; CR, complete response; ORR, objective response rate; DCR, disease control rate.

**Figure 1:**
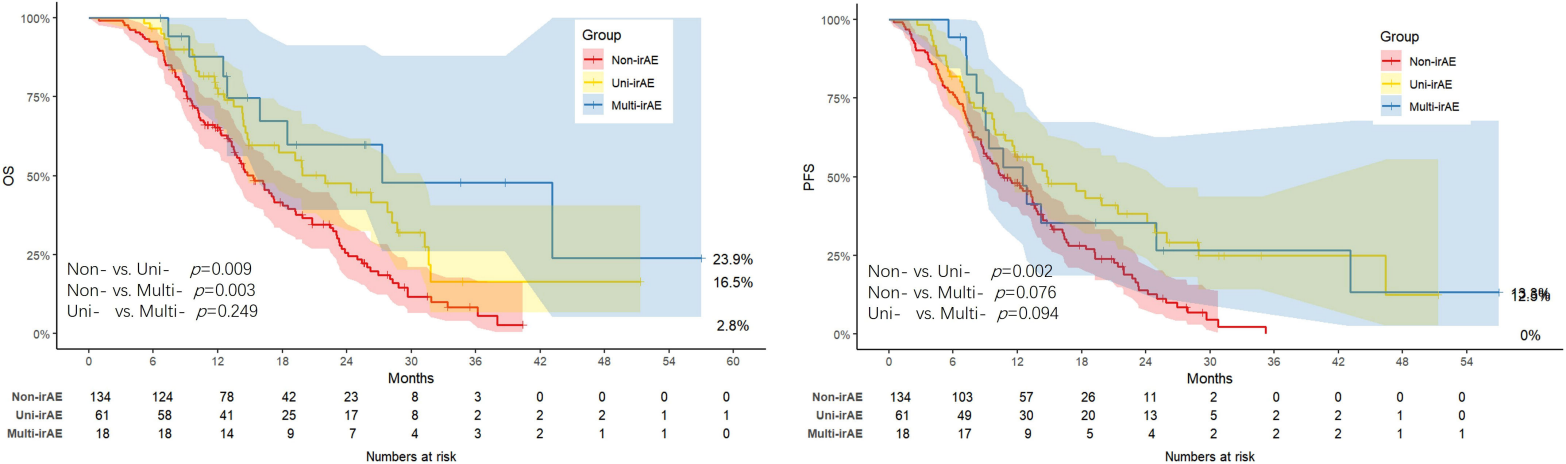
The Kaplan-Meier curve of progression-free survival (PFS) and overall survival (OS) of all patients.

The median PFS was 10.67 months (95% confidence interval [CI]: 8.83-13.67) in the non-irAE group, 14.87 months (95% CI: 11.53-24.83) in the uni-irAE group, and 12.47 months (95% CI: 7.68-17.25) in the multi-irAEs group, respectively (**Supplementary Table S3**). Kaplan-Meier analysis (**Figure 1**) revealed that the uni-irAE group experienced a significantly longer median PFS compared to the non-irAE group (*p* = 0.002), while no significant differences were found between the uni-irAE and multi-irAEs groups (*p* = 0.094) or between the non-irAE and multi-irAEs groups (*p* = 0.076). Multivariate Cox analysis confirmed uni-irAE as an independent protective factor for PFS (hazard ratio [HR]: 0.57; 95% CI: 0.39-0.83; *p* = 0.003), alongside other independent predictors such as chemotherapy regimen (HR: 0.56; 95% CI: 0.38-0.85; *p* = 0.006), ECOG score (HR: 2.11;95% CI: 1.42-3.14; *p* < 0.001), and number of metastases (HR: 1.63;95% CI: 1.15-2.29; *p* = 0.005) (**Table 3**). These findings collectively indicate that the occurrence of uni-irAE is associated with superior PFS.

**Table 3:** Univariate and multiple Cox proportional hazard model for PFS in ESCC patients.

Variables	Univariate analysis		Multivariate analysis	
	HR (95%CI)	*P-*value	HR (95%CI)	*P-*value
Gender				
Female	1.00 (Reference)			
Male	1.10 (0.78-1.54)	0.595		
Age group				
<65	1.00 (Reference)			
≥65	0.93 (0.67-1.29)	0.659		
ECOG				
≤1	1.00 (Reference)		1.00 (Reference)	
>1	2.23 (1.52-3.29)	<.001	2.11 (1.42-3.14)	<.001
NLR				
NLR<3	1.00 (Reference)			
NLR≥3	1.11 (0.81-1.52)	0.533		
TNM stage				
II	1.00 (Reference)			
III	1.49 (0.83-2.66)	0.182		
IV	1.70 (0.97-3.00)	0.065		
Tumor differentiation				
Medium to high	1.00 (Reference)			
Low	1.08 (0.72-1.62)	0.721		
Tumor site				
Upper	1.00 (Reference)			
Middle	0.88 (0.57-1.36)	0.561		
Lower	0.84 (0.53-1.34)	0.470		
Other	0.74 (0.46-1.19)	0.215		
PD-L1 expression				
Negative	1.00 (Reference)			
Positive	1.15 (0.63-2.10)	0.638		
Unknown	1.49 (0.87-2.56)	0.150		
History of surgery				
No	1.00 (Reference)			
Yes	1.01 (0.57-1.78)	0.976		
Number of metastases				
≤1	1.00 (Reference)		1.00 (Reference)	
>1	1.61 (1.15-2.25)	0.006	1.63 (1.15-2.29)	0.005
Chemotherapy regimen				
Others	1.00 (Reference)		1.00 (Reference)	
TP	0.54 (0.36-0.80)	0.002	0.56 (0.38-0.85)	0.006
IrAE groups				
Non-irAE	1.00 (Reference)		1.00 (Reference)	
Uni-irAE	0.57 (0.39-0.82)	0.003	0.57 (0.39-0.83)	0.003
Multi-irAEs	0.61 (0.34-1.10)	0.100	0.62 (0.33-1.13)	0.118

IrAEs, immune-related adverse events; ESCC, esophageal squamous cell carcinoma; Uni-irAE, single-organ irAE; Multi-irAEs, multiple-organ irAEs; NLR, Neutrophil to Lymphocyte Ratio; ECOG, Eastern Cooperative Oncology Group; TNM, tumor-node-metastasis; HR, hazard ratio; PD-L1, programmed cell death ligand 1; PFS, progression free survival.

The median OS was 15.27 months (95% CI: 13.53-18.50) in the non-irAE group, 22.00 months (95% CI: 14.77-31.23) in the uni-irAE group, and 27.27 months (95% CI: 15.87-47.27) in the multi-irAEs group, respectively (**Supplementary Table S3**). Kaplan-Meier analysis revealed that the median OS was significantly longer in both the uni-irAE (*p* = 0.009) and multi-irAEs (*p* = 0.003) groups compared to the non-irAE group. However, no significant difference in OS was observed between the uni-irAE and multi-irAEs groups(*p* = 0.249). Then multivariate Cox analysis identified both uni-irAE (HR: 0.64; 95% CI: 0.44-0.95; *p* = 0.028) and multi-irAEs (HR: 0.41; 95% CI: 0.20-0.86; *p* = 0.019) as independent protective factors for OS. In contrast, a higher ECOG score and an increased number of metastases were identified as independent predictors of shorter OS (**Table 4**). Collectively, these findings demonstrate that the occurrence of irAEs modify the treatment efficiency of ICI with uni-irAE linking with higher DCR, PFS, and OS while multi-irAEs linking to the most favorable OS.

**Table 4:** Univariate and multiple Cox proportional hazard model for OS in ESCC patients.

Variables	Univariate analysis		Multivariate analysis	
	HR (95%CI)	*P-*value	HR (95%CI)	*P-*value
Gender				
Female	1.00 (Reference)			
Male	1.18 (0.82-1.69)	0.383		
Age group				
<65	1.00 (Reference)			
≥65	0.92 (0.65-1.29)	0.623		
ECOG				
≤1	1.00 (Reference)		1.00 (Reference)	
>1	2.15 (1.43-3.24)	<.001	2.02 (1.34-3.06)	<.001
NLR				
NLR<3	1.00 (Reference)			
NLR≥3	1.29 (0.92-1.80)	0.142		
TNM stage				
II	1.00 (Reference)			
III	1.00 (0.55-1.81)	0.996		
IV	1.27 (0.72-2.25)	0.409		
Tumor differentiation				
Medium to high	1.00 (Reference)			
Low	0.79 (0.50-1.25)	0.316		
Tumor site				
Upper	1.00 (Reference)			
Middle	1.00 (0.63-1.59)	0.993		
Lower	1.00 (0.61-1.64)	0.989		
Other	0.87 (0.53-1.43)	0.575		
PD-L1 expression				
Negative	1.00 (Reference)			
Positive	0.97 (0.51-1.86)	0.935		
Unknown	1.34 (0.75-2.39)	0.329		
History of surgery				
No	1.00 (Reference)			
Yes	1.02 (0.57-1.81)	0.955		
Number of metastases				
≤1	1.00 (Reference)		1.00 (Reference)	
>1	1.75 (1.23-2.50)	0.002	1.74 (1.22-2.47)	0.002
Chemotherapy regimen				
Others	1.00 (Reference)			
TP	0.68 (0.45-1.02)	0.060		
IrAE groups				
Non-irAE	1.00 (Reference)		1.00 (Reference)	
Uni-irAE	0.59 (0.40-0.87)	0.008	0.64 (0.44-0.95)	0.028
Multi-irAEs	0.36 (0.17-0.74)	0.006	0.41 (0.20-0.86)	0.019

IrAEs, immune-related adverse events; ESCC, esophageal squamous cell carcinoma; Uni-irAE, single-organ irAE; Multi-irAEs, multiple-organ irAEs; HR, hazard ratio; NLR, Neutrophil to Lymphocyte Ratio; ECOG, Eastern Cooperative Oncology Group; TNM, tumor-node-metastasis; PD-L1, programmed cell death ligand 1; OS, overall survival.

### 3.3 Co-occurrence pattern of multi-irAEs

**Figure 2:**
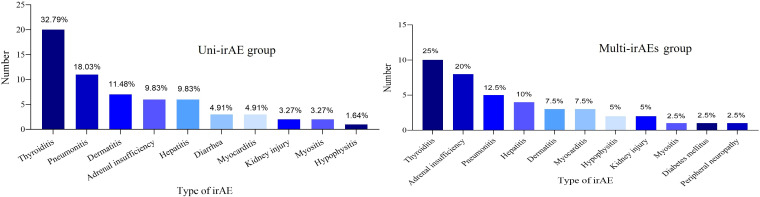
Types of irAEs in the uni-irAE and multi-irAE groups.

The most common uni-irAE was thyroiditis (32.79%), while the most frequent multi-irAEs combinations were thyroiditis-adrenal insufficiency (16.67%) (**Supplementary Table S4**; **Figure 2**). Notably, most multi-irAEs developed sequentially (77.78%) rather than simultaneously. Univariate logistic regression analysis showed that endocrine irAEs were significantly associated with the occurrence of multi-irAEs (odds ratio [OR]:3.27; 95% CI: 1.04-10.32; *p* = 0.043). In contrast, hepatitis, dermatologic, and pulmonary irAEs were not significantly associated with the development of multi-irAEs (all *p* > 0.05) (**Supplementary Table S5**). We explored potential predictors of multi-irAEs using logistic regression, but none of the patient characteristics reached statistical significance (data not shown).

### 3.4 Timepoint of irAEs onset

**Figure 3:**
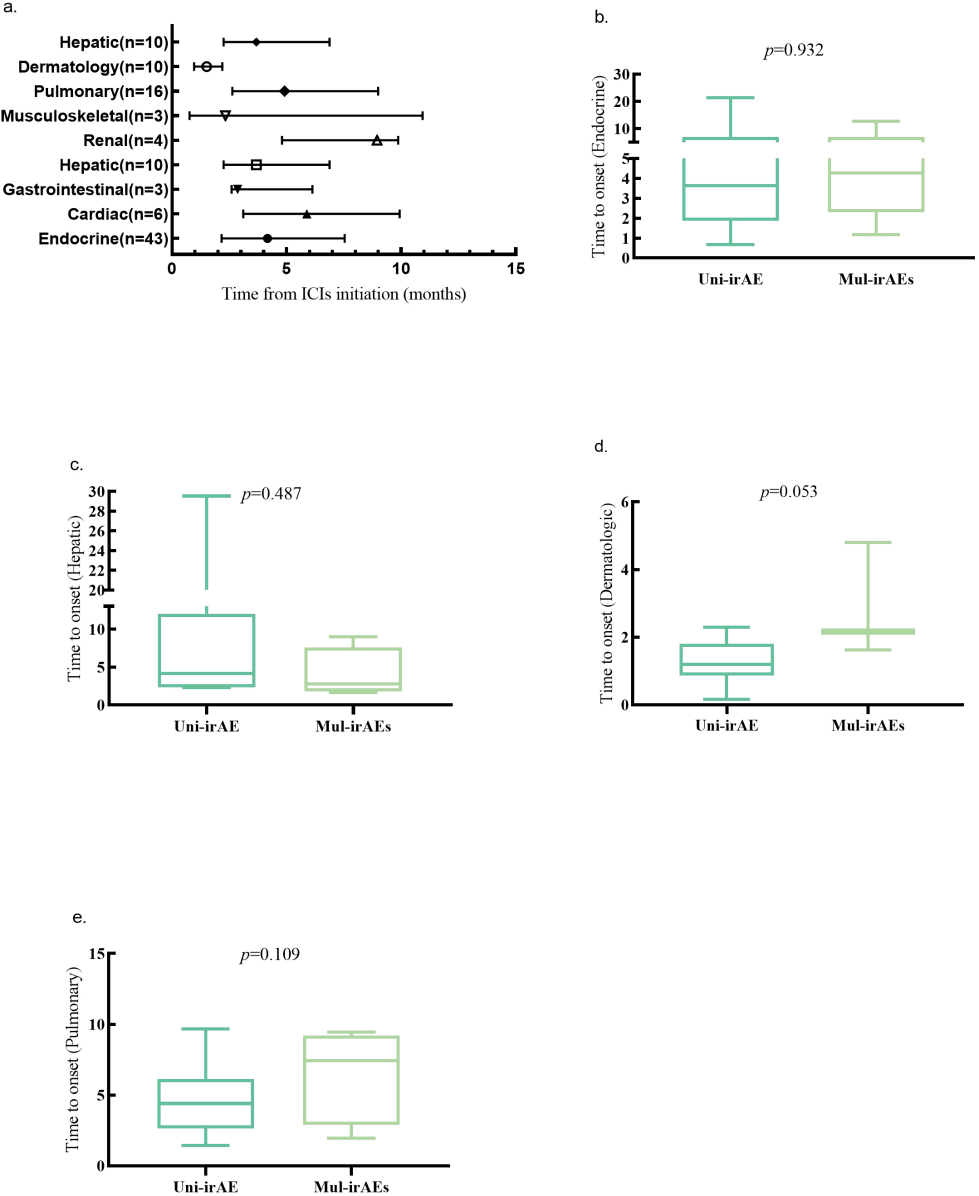
**(a)** Time to onset of organ-specific irAEs (median, range, and months). Time to onset of endocrine **(b)**, hepatic **(c)**, dermatologic **(d)**, pulmonary **(e)** irAEs in uni-irAE and multi-irAE groups.

As shown in **Figure 3**, the median times to onset for the four organ irAEs were 1.90 months (dermatologic irAEs), 4.17 months (endocrine irAEs), 3.68 months (hepatic irAEs), and 4.92 months (pulmonary irAEs). A comparison of onset times of the same irAE between uni-irAE and multi-irAEs groups revealed no significant differences for these irAE category (*p*>0.05). This suggests that the development of multiple irAEs is not a time-dependent phenomenon.

### 3.5 Effect of organ-specific irAEs on ICI outcomes

We analyzed the impact of organ-specific irAEs on treatment efficacy and prognosis. Endocrine irAEs uniquely predicted better therapeutic efficacy and survival. Patients developing these irAEs showed a higher DCR (100% *vs*. 83.8%, *p* = 0.006) and significantly longer median PFS (18.33 months *vs*. 10.67 months, *p* < 0.001) and OS (24.37 months *vs*. 15.33 months, *p* = 0.006) (**Supplementary Tables S6**, **S7**; **Supplementary Figure S1**). Furthermore, Multivariate analysis identified endocrine irAEs as an independent protective factor for both extended PFS (HR: 0.47; 95% CI: 0.29-0.78; *p* = 0.003) and OS (HR: 0.55; 95% CI: 0.32-0.92; *p* = 0.024) (**Tables 5**, **6**). No other organ-specific irAEs had a statistically significant impact on prognosis (all *p* > 0.05) (**Supplementary Tables S8**, **S9**).

**Table 5:** Univariate and multivariate Cox proportional hazards analysis of PFS by endocrine irAE.

Variables	Univariate analysis		Multivariate analysis	
	HR (95%CI)	*P*-value	HR (95%CI)	*P*-value
Gender				
Female	1.00 (Reference)			
Male	1.10 (0.78 - 1.54)	0.595		
Age group				
<65	1.00 (Reference)			
≥65	0.93 (0.67 - 1.29)	0.659		
ECOG				
≤1	1.00 (Reference)		1.00 (Reference)	
>1	2.23 (1.52 - 3.29)	<.001	2.15 (1.45 - 3.20)	<.001
NLR				
NLR<3	1.00 (Reference)			
NLR≥3	1.11 (0.81 - 1.52)	0.533		
TNM stage				
II	1.00 (Reference)			
III	1.49 (0.83 - 2.66)	0.182		
IV	1.70 (0.97 - 3.00)	0.065		
Tumor differentiation				
Medium to high	1.00 (Reference)			
Low	1.08 (0.72 - 1.62)	0.721		
Tumor site				
Upper	1.00 (Reference)			
Middle	0.88 (0.57 - 1.36)	0.561		
Lower	0.84 (0.53 - 1.34)	0.470		
Other	0.74 (0.46 - 1.19)	0.215		
PD-L1 expression				
Negative	1.00 (Reference)			
Positive	1.15 (0.63 - 2.10)	0.638		
Unknown	1.49 (0.87 - 2.56)	0.150		
History of surgery				
No	1.00 (Reference)			
Yes	1.01 (0.57 - 1.78)	0.976		
Number of metastases				
≤1	1.00 (Reference)		1.00 (Reference)	
>1	1.61 (1.15 - 2.25)	0.006	1.56 (1.11 - 2.19)	0.011
Chemotherapy regimen				
Others	1.00 (Reference)		1.00 (Reference)	
TP	0.54 (0.36 - 0.80)	0.002	0.62 (0.42 - 0.93)	0.020
Endocrine irAEs				
Others	1.00 (Reference)		1.00 (Reference)	
Endocrine irAEs	0.43 (0.26 - 0.70)	<.001	0.47 (0.29 - 0.78)	0.003

IrAEs, immune-related adverse events; ESCC, esophageal squamous cell carcinoma; HR, hazard ratio; NLR, Neutrophil to Lymphocyte Ratio; ECOG, Eastern Cooperative Oncology Group; TNM, tumor-node-metastasis; PD-L1, programmed cell death ligand 1; PFS, progression free survival.

**Table 6:** Univariate and multivariate Cox proportional hazards analysis of OS by endocrine irAE.

Variables	Univariate analysis		Multivariate analysis	
	HR (95%CI)	*P-*value	HR (95%CI)	*P-*value
Gender				
Female	1.00 (Reference)			
Male	1.18 (0.82 - 1.69)	0.383		
Age group				
<65	1.00 (Reference)			
≥65	0.92 (0.65 - 1.29)	0.623		
ECOG				
≤1	1.00 (Reference)		1.00 (Reference)	
>1	2.15 (1.43 - 3.24)	<.001	2.15 (1.42 - 3.24)	<.001
NLR				
NLR<3	1.00 (Reference)			
NLR≥3	1.29 (0.92 - 1.80)	0.142		
TNM stage				
II	1.00 (Reference)			
III	1.00 (0.55 - 1.81)	0.996		
IV	1.27 (0.72 - 2.25)	0.409		
Tumor differentiation				
Medium to high	1.00 (Reference)			
Low	0.79 (0.50 - 1.25)	0.316		
Tumor site				
Upper	1.00 (Reference)			
Middle	1.00 (0.63 - 1.59)	0.993		
Lower	1.00 (0.61 - 1.64)	0.989		
Other	0.87 (0.53 - 1.43)	0.575		
PD-L1 expression				
Negative	1.00 (Reference)			
Positive	0.97 (0.51 - 1.86)	0.935		
Unknown	1.34 (0.75 - 2.39)	0.329		
History of surgery				
No	1.00 (Reference)			
Yes	1.02 (0.57 - 1.81)	0.955		
Number of metastases				
≤1	1.00 (Reference)		1.00 (Reference)	
>1	1.75 (1.23 - 2.50)	0.002	1.74 (1.22 - 2.49)	0.002
Chemotherapy regimen				
Others	1.00 (Reference)			
TP	0.68 (0.45 - 1.02)	0.060		
Endocrine irAEs				
Others	1.00 (Reference)		1.00 (Reference)	
Endocrine irAEs	0.46 (0.27 - 0.79)	0.005	0.55 (0.32 - 0.92)	0.024

IrAEs, immune-related adverse events; ESCC, esophageal squamous cell carcinoma; HR, hazard ratio; NLR, Neutrophil to Lymphocyte Ratio; ECOG, Eastern Cooperative Oncology Group; TNM, tumor-node-metastasis; PD-L1, programmed cell death ligand 1; OS, overall survival.

### 3.6 Effect of severity of irAEs on ICI outcomes

**Figure 4:**
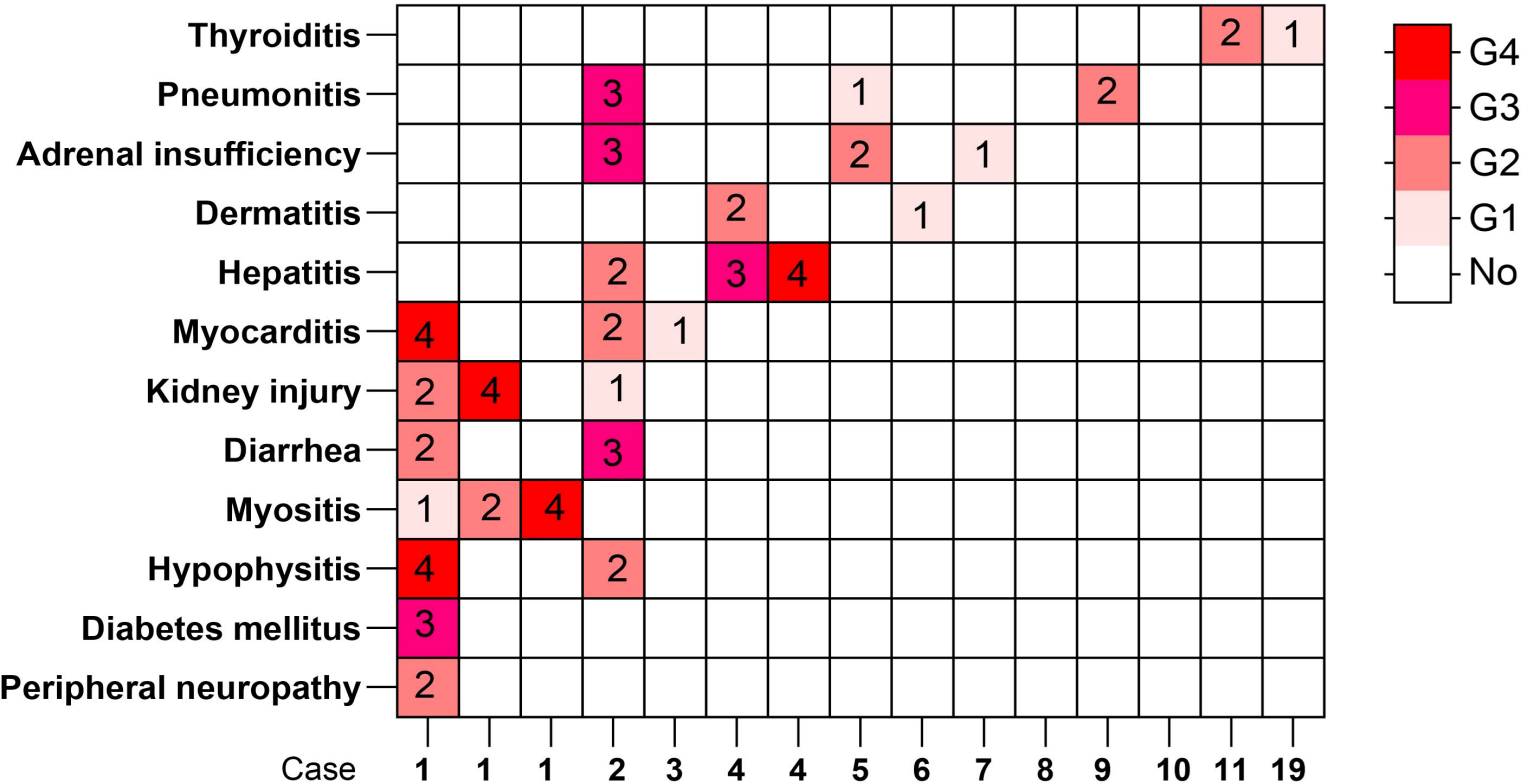
Distribution of irAEs by severity grade.

The number of adverse events for each severity grade is shown in **Figure 4**. We divided the patients into non-irAE, mild-irAE, and severe-irAE groups according to irAE severity, and subsequently analyzed their therapeutic efficacy and prognosis. The results showed that patients with mild irAEs tended to have better DCR, PFS, and OS than those without irAEs (DCR: 95.2% *vs*. 82.1%, *p* = 0.012; PFS: 17.50 months *vs*. 10.67 months; *p* < 0.001; OS: 22.00 months *vs*. 15.27 months; *p* < 0.001) (**Supplementary Table S10**, **11**). Multivariate analysis revealed that mild-irAE was an independent protective factor for both PFS and OS(PFS: HR = 0.53; 95% CI: 0.36-0.78; *p* = 0.001; OS: HR = 0.54; 95% CI: 0.36-0.82; *p* = 0.004), whereas no such association was observed for severe irAEs (*p*>0.05) (**Supplementary Tables S12**, **13**).

## 4 Discussion

The occurrence of irAEs appears to be a promising predictive biomarker for ICI efficacy across various cancer type (19, 20, 23, 25–34). Previous studies have demonstrated that in patients with advanced EC, the development of irAEs was associated with significantly improved outcomes (23, 36). We performed a deeper analysis that found a more nuanced relationship, which reveals that uni-irAE served as an independent protective factor for both PFS and OS. Multi-irAEs were also independently associated with improved OS, though their benefit for PFS did not reach statistical significance possibly due to limited follow-up or sample size. Additionally, no significant difference in survival was observed between the uni-irAE and multi-irAEs groups. Overall, the occurrence of both uni-irAE and multi-irAEs predicted superior survival, consistent with findings in other malignancies (23, 27, 40–42).

The positive correlation between irAEs and treatment efficacy is thought to stem from shared immunological pathways. One such pathway is T cell cross-reactivity against shared antigens, supported by the discovery of identical T cell receptor (TCR) sequences in both tumors and irAE-affected tissues (43–49). Concurrently, cytokines play a significant role in both inducing irAEs and eliminating tumor cells. Patients with irAEs exhibit elevated levels of cytokines, such as Interferon-gamma (IFN-γ) and Interleukin-7 (IL-7), which are associated with both autoimmune toxicity and a favorable therapeutic response (50–55). Furthermore, ICI-induced depletion of regulatory T cells (Tregs) can unleash autoreactive T cells, simultaneously causing irAEs and enhancing anti-tumor responses (48, 56, 57). Finally, the composition of the gut microbiota is also known to shape both therapeutic efficacy and the likelihood of irAEs. Mechanistically, specific bacterial signatures can modulate systemic immunity and lower the T-cell activation threshold, thereby enhancing anti-tumor responses while simultaneously increasing susceptibility to autoimmune toxicity (44, 58–63). The prognostic value of multi-irAEs varies significantly across cancer types, being superior in gastric, small cell lung cancer, and renal cell carcinoma, but not in non-small cell lung cancer (23, 25–28). In our ESCC cohort, multi-irAEs were associated with the longest OS but an intermediate PFS, which was shorter than that of the uni-irAE group but longer than that of the non-irAE group. The development of multi-irAEs suggests a more robust systemic immune response or a profoundly altered tumor microenvironment that better facilitates immunotherapy (64).This heightened immune reactivity is believed to be the biological basis for the “tail effect,” which enables long-term survival even after initial progression (65). Conversely, the intermediate PFS may be attributable to the heterogeneity of tumor types or the limited sample size. Therefore, further prospective trials and immunological studies are warranted to delineate these complex relationships.

Our deeper analysis of irAEs revealed that co-occurrence patterns and severity are key prognostic factors, shifting the focus from a binary view to a more nuanced understanding.

The most frequent co-occurrences were thyroid-adrenal and thyroid-myocarditis pairs, and the time to onset for any irAE was similar between uni-irAE and multi-irAEs groups, indicating that the development of multiple irAEs is not time-dependent. While endocrine irAEs predicted the development of multi-irAEs, they were also associated with superior survival outcomes. Regarding the clinical timing of these prognostic markers, in some cases, such as skin irAE, it can serve as a clinical response marker earlier than the first imaging evaluation because its average time of onset (1.9 months) precedes the first imaging assessment. In other cases, such as hepatic (3.68 months), endocrine (4.17 months), and pulmonary toxicity (4.92 months), their average time of occurrence is indeed later than the first imaging evaluation. However, traditional imaging evaluation can no longer fully and accurately reflect the true efficacy of immunotherapy, particularly in tumors where cancer cell reduction and corresponding lymphocyte infiltration occur without changes in tumor volume. Therefore, even irAEs that occur later than imaging evaluation still possess auxiliary predictive functions.

The underlying mechanisms for irAE co-occurrence and the positive prognostic impact of endocrine irAEs remain unclear, but several mechanisms have been proposed. One proposed mechanism for multi-organ co-occurrence is a systemic, uncontrolled immune response, such as a cytokine storm, which can cause simultaneous damage across different systems (66–68). B cell activation may be another mechanism, as it elevates autoantibodies that can deposit in specific organs and trigger inflammation. For example, patients with hepatic irAEs showed increased levels of anti-thyroglobulin Immunoglobulin G (IgG), a B cell-derived antibody that can precipitate thyroid irAEs (19). The survival advantage of endocrine irAEs may stems from two key factors. First, given that endocrine tissues are known to exhibit intense lymphocyte and cytokine infiltration, they become a primary target in this systemic assault, leading to the onset of endocrine irAEs (69, 70). Second, their typically low-grade nature allows for management with gentle therapies, avoiding high-dose corticosteroids that would suppress the beneficial immune activity. A small sample size may explain the inconclusive findings for hepatic, pulmonary, and gastrointestinal irAEs.

Analysis of irAE severity revealed that mild irAEs were associated with superior DCR, PFS, and OS compared to non-irAE in ESCC patients, which corroborated early findings in lung cancer and renal cell carcinoma (42, 71–73). The prognosis of severe irAEs, however, is controversial; while some studies link them to the worst outcomes, others show no difference from non-irAE (74). In our cohort, severe irAEs showed a trend toward reduced OS when compared to mild irAEs, but they displayed the extension of OS than that of non-irAE. The inferior prognosis of severe irAEs compared to mild irAEs may be attributed to two primary factors. First, ICI treatment is often discontinued following the onset of severe irAEs, potentially limiting the cumulative benefits of continued therapy (75). Second, the high-dose corticosteroids required to manage severe irAEs may compromise the efficacy of immunotherapy (76–83). Then, the favorable prognosis of patients with severe irAEs comparing to the non-irAE group may be explained by the following mechanisms. First, the onset of any irAE can serve as a biomarker for a robust therapeutic response, as discussed previously. This enhanced immunity is thought to drive the “tail effect,” a phenomenon where the immune system continues to exert an anti-tumor effect even after treatment cessation (65, 84). Second, close clinical monitoring and prompt intervention for irAEs can prevent severe events from becoming life-threatening, thereby improving overall outcomes. Future studies with larger sample sizes are warranted to validate these findings.

The impact of glucocorticoids (GCs) on the efficacy of immunotherapy remains a subject of debate, potentially influenced by complex interplays between dosage, treatment duration, and administration timing. In this study, after excluding non-immunosuppressive doses (such as antiemetic dexamethasone or replacement hydrocortisone), the duration of GC treatment ranged from 7 to 73 days in 17 irAE patients. ROC analysis (AUC = 0.65) identified the GC treatment time longer than 54.5-day associated with shorter Median OS (8.65 *vs*. 28.17 months, *p* = 0.006) in these ESCC patients. The Median PFS also reflected a trend of harm associated with prolonged steroid use, although statistically non-significant (7.98 *vs*. 11.08 months, *p* = 0.115) (data not shown).

Currently, research on the prognostic impact of GCs primarily focuses on administration timing and dosage. Regarding timing, baseline use of high-dose GCs is often associated with poorer outcomes, likely reflecting baseline characteristics such as high tumor burden or poor performance status (80, 85, 86). In contrast, therapeutic intervention during the induction phase, typically the first 4 to 8 weeks, remains a subject of debate. Some studies suggest that early immunosuppression may impair long-term efficacy by interfering with T-cell clonal expansion and priming, potentially abrogating the initial anti-tumor immune response (81, 87). Conversely, other evidence indicates that patients requiring corticosteroids for early-onset irAEs still exhibit superior survival outcomes compared to those steroid naïve group (88).Subsequent large-scale inquiries and meta-analyses are still needed. Furthermore, the duration of the tapering period is critical; rapid tapering of less than 4–6 weeks can easily trigger irAE rebound, leading to cumulative harm from secondary high-dose exposure (83, 89). In our analysis, 54.5 days (approximately 8 weeks) emerged as a critical threshold for treatment duration. Finally, concerning dosage, evidence suggests that the peak dose is more immunologically disruptive than the cumulative dose, as ultra-high concentrations can induce lymphocyte apoptosis, leading to poorer outcomes (79, 90, 91). Therefore, in clinical practice, clinicians should aim to balance effective toxicity control with antitumor efficacy. We propose managing the intervention timing and tapering schedule to keep the total duration ideally within this 54.5-day window, while also carefully considering peak dosage to minimize immunological disruption.

This study has several limitations. First, its retrospective, single-center design and relatively small sample size limit our ability to account for confounding variables and increase the risk of selection bias. Second, variations in patient treatment regimens could have influenced the final outcomes. Third, the short follow-up period for some patients and the presence of censored data may have led to an incomplete assessment of median survival times. Considering these limitations, future follow-up studies, multi-center collaborations and prospective trials are warranted to enhance the reliability and validity of these findings.

## 5 Conclusion

The occurrence of both uni-irAE and multi-irAEs was associated with favorable prognosis in ESCC patients treated with ICIs. Furthermore, patients who developed endocrine irAEs or mild irAEs also demonstrated improved efficacy, suggesting their potential as clinical response markers for a positive response to therapy. This finding emphasizes the necessity of vigilant monitoring and early intervention for irAEs in patients undergoing ICIs.

## Data Availability

The datasets presented in this article are not readily available because The datasets generated during this study are not publicly available due to ethical restrictions and patient privacy concerns. However, the data can be made available from the corresponding author upon reasonable request, subject to approval by the institutional ethics committee. Requests to access the datasets should be directed to TZ, thzhang8116@aliyun.com.

## Supplementary material

Supplementary Table 1 Treatment regimen of patients in ESCC patients.

Supplementary Table 2 Comparison of irAE Incidence Among Different Types of ICIs.

Supplementary Table 3 Median PFS and OS of non-irAE, uni-irAE, and multi-irAEs groups in ESCC patients.

Supplementary Table 4 The irAE cases number of uni-irAE and multi-irAEs groups in ESCC patients.

Supplementary Table 5 Univariate logistic regression analysis of the relationship between each organ-specific irAE and multi-irAEs.

Supplementary Table 6 Response rate analysis of each organ-specific irAE vs. other patients.

Supplementary Table 7 Median PFS and OS of each organ-specific group vs. other patients.

Supplementary Table 8 Univariate Cox analysis of organ-specific irAEs and PFS in ESCC patients.

Supplementary Table 9 Univariate Cox analysis of organ-specific irAEs and OS in ESCC patients.

Supplementary Table 10 Response to ICI of irAEs, non-irAE, mild-irAE, and severe-irAE groups in ESCC patients.

Supplementary Table 11 Median PFS and OS of non-irAE, mild-irAE, and severe-irAE groups in ESCC patients.

Supplementary Table 12 Univariate and Multivariate Cox Proportional Hazards Analysis of PFS by irAE severity.

Supplementary Table 13 Univariate and Multivariate Cox Proportional Hazards Analysis of OS by irAE severity.

Supplementary Figure 1 **(A-J)** The Kaplan-Meier curve of progression-free survival (PFS) and overall survival (OS) of each specific irAE with a case number of more than 10 patients in ESCC patients. **(A)** PFS of endocrine irAE patients. **(B)** OS of endocrine irAE patients. **(C)** PFS of dermatologic irAE patients. **(D)** OS of dermatologic irAE patients. **(E)** PFS of hepatic irAE patients. **(F)** OS of hepatic irAE patients. **(G)** PFS of pulmonary irAE patients. **(H)** OS of pulmonary irAE patients.
