# Supplementary material for: Impact of immune-related adverse events on treatment outcomes in advanced esophageal squamous cell carcinoma treated with immune checkpoint inhibitors
Source: Front Immunol. 2026 Feb 5;17:1741482. doi: 10.3389/fimmu.2026.1741482 (PMC12916662; doi:10.3389/fimmu.2026.1741482)
Supplement: Supplementary Table 1 — Treatment regimen of patients in ESCC patients. [file DataSheet1.docx]

Table S1. Treatment regimen of patients in ESCC patients.

| No. of patients | Therapeutic schedule | |
| --- | --- | --- |
|  | ICIs | Chemotherapeutic regimen |
| 9 | Pembrolizumab | Paclitaxel＋Platinum compound |
| 1 | Pembrolizumab | Paclitaxel |
| 65 | Sintilimab | Paclitaxel＋Platinum compound |
| 4 | Sintilimab | Paclitaxel |
| 2 | Sintilimab | - |
| 1 | Sintilimab | Platinum compound |
| 1 | Sintilimab | Platinum compound＋S-1 |
| 2 | Sintilimab | Platinum compound＋5-FU |
| 2 | Sintilimab | Platinum compound＋Docetaxel |
| 70 | Camrelizumab | Paclitaxel＋Platinum compound |
| 2 | Camrelizumab | - |
| 5 | Camrelizumab | Paclitaxel |
| 1 | Camrelizumab | Gemcitabine |
| 1 | Camrelizumab | Platinum compound＋5-FU |
| 30 | Tislelizumab | Paclitaxel＋Platinum compound |
| 5 | Tislelizumab | Paclitaxel |
| 1 | Tislelizumab | - |
| 1 | Tislelizumab | S-1 |
| 2 | Serplulimab | Paclitaxel＋Platinum compound |
| 1 | Nivolumab | Paclitaxel＋Platinum compound |
| 5 | Toripalimab | Paclitaxel＋Platinum compound |
| 1 | Toripalimab | Paclitaxel |
| 1 | Toripalimab | Platinum compound＋S-1 |

IrAEs, immune-related adverse events; ESCC, esophageal squamous cell carcinoma.

| Type of irAEs | Type of ICIs | | | |  |
| --- | --- | --- | --- | --- | --- |
|  | Pembrolizumab  n=6 | Sintilimab  n=19 | Camrelizumab  n=32 | Tislelizumab  n=16 | p-value |
| Endorine | 5 | 12 | 17 | 5 | 0.120 |
| Thyroiditis | 3 | 9 | 14 | 3 | 0.277 |
| Adrenal insufficiency | 2 | 3 | 4 | 3 | 0.579 |
| Hypophysitis | 1 | 1 | 0 | 1 | 0.098 |
| Myocarditis | 1 | 1 | 2 | 0 | 0.368 |
| Diarrhea | 0 | 0 | 1 | 2 | 0.397 |
| Hepatitis | 0 | 2 | 3 | 4 | 0.420 |
| Dermatitis | 1 | 1 | 5 | 2 | 0.668 |
| Pneumonitis | 1 | 4 | 6 | 4 | 0.970 |
| Kidney injury | 0 | 0 | 3 | 0 | 0.476 |
| Myositis | 0 | 1 | 1 | 1 | 0.999 |

Table S2. Comparison of irAE Incidence Among Different Types of ICIs.

IrAEs, immune-related adverse events; ICIs, immune checkpoint inhibitors.

Table S3. Median PFS and OS of non-irAE, uni-irAE, and multi-irAEs groups in ESCC patients.

| Covariate | Median PFS (95% CI)  (months) | *p-*value  (Log-rank) | Median OS (95% CI)  (months) | *p-*value  (Log-rank) |
| --- | --- | --- | --- | --- |
| Non-irAE group vs irAEs group |  | 0.001 |  | <.001 |
| Non-irAE group | 10.67 (8.83 - 13.67) |  | 15.27 (13.53 - 18.50) |  |
| irAEs group | 14.20 (10.83 - 24.13) |  | 24.37 (17.67 - 31.23) |  |
| Non-irAE group vs Uni-irAE group |  | 0.002 |  | 0.009 |
| Non-irAE group | 10.67 (8.83 - 13.67) |  | 15.27 (13.53 - 18.50) |  |
| Uni-irAE group | 14.87 (11.53 - 24.83) |  | 22.00 (14.77 - 31.23) |  |
| Non-irAE group vs Multi -irAEs group |  | 0.076 |  | 0.003 |
| Non-irAE group | 10.67 (8.83 - 13.67) |  | 15.27 (13.53 - 18.50) |  |
| Multi -irAEs group | 12.47 (7.68 – 17.25) |  | 27.27 (15.87 – 47.27) |  |
| Uni-irAE group vs Multi -irAEs group |  | 0.094 |  | 0.249 |
| Uni-irAE group | 14.87 (11.53 - 24.83) |  | 22.00 (14.77 - 31.23) |  |
| Multi -irAEs group | 12.47 (7.68 - 17.25) |  | 27.27 (15.87 - 47.27) |  |

IrAEs, immune-related adverse events; Uni-irAE, single-organ irAE; Multi-irAEs, multiple-organ irAEs; OS, overall survival; PFS, progression free survival; ESCC, esophageal squamous cell carcinoma.

Table S4. The irAE cases number of uni-irAE and multi-irAEs groups in ESCC patients.

| Covariate | N (number) | % (percentage) |
| --- | --- | --- |
| Uni-irAE(n=61) |  |  |
| Thyroiditis | 20 | 32.79 |
| Adrenal insufficiency | 6 | 9.83 |
| Hypophysitis | 1 | 1.64 |
| Myocarditis | 3 | 4.91 |
| Diarrhea | 3 | 4.91 |
| Hepatitis | 6 | 9.83 |
| Dermatitis | 7 | 11.48 |
| Pneumonitis | 11 | 18.03 |
| Kidney injury | 2 | 3.27 |
| Myositis | 2 | 3.27 |
| Multi-irAEs(n=18) |  |  |
| Thyroiditis-Adrenal insufficiency | 3 | 16.67 |
| Thyroiditis-Hypophysitis | 2 | 11.11 |
| Thyroiditis-Myocarditis | 2 | 11.11 |
| Thyroiditis-Pneumonitis | 1 | 5.56 |
| Thyroiditis-Adrenal insufficiency-Dermatitis | 1 | 5.56 |
| Thyroiditis-Adrenal insufficiency-Hepatitis | 1 | 5.56 |
| Adrenal insufficiency-Dermatitis | 1 | 5.56 |
| Adrenal insufficiency-Pneumonitis | 1 | 5.56 |
| Adrenal insufficiency-Diabetes mellitus-Hepatitis | 1 | 5.56 |
| Pneumonitis-Dermatitis | 1 | 5.56 |
| Pneumonitis-Myocarditis | 1 | 5.56 |
| Hepatitis-Kidney injury | 1 | 5.56 |
| Hepatitis-Kidney injury-Pneumonitis | 1 | 5.56 |
| Myositis-Peripheral neuropathy | 1 | 5.56 |

IrAEs, immune-related adverse events; ESCC, esophageal squamous cell carcinoma; Uni-irAE, single-organ irAE; Multi-irAEs, multiple-organ irAEs

Table S5. Univariate logistic regression analysis of the relationship between each organ-specific irAE and multi-irAEs.

| Covariate | Univariate Analysis | |
| --- | --- | --- |
|  | OR (95% CI) | *p*-value |
| Endocrine irAEs |  |  |
| Others | 1.00 (Reference) |  |
| Endocrine irAEs | 3.27 (1.04 - 10.32) | 0.043 |
| Dermatologic irAEs |  |  |
| Others | 1.00 (Reference) |  |
| Dermatologic irAEs | 1.54 (0.36 - 6.70) | 0.563 |
| Hepatic irAEs |  |  |
| Others | 1.00 (Reference) |  |
| Hepatic irAEs | 2.62 (0.65 - 10.56) | 0.176 |
| Pulmonary irAEs |  |  |
| Others | 1.00 (Reference) |  |
| Pulmonary irAEs | 1.75 (0.52 - 5.93) | 0.370 |

IrAEs, immune-related adverse events; OR, odds ratio; CI, confidence interval; Multi-irAEs, multiple-organ irAEs. Other patients, including all the patients except the corresponding organ-specific irAE patients;

Table S6. Response rate analysis of each organ-specific irAE vs. other patients.

| Comparison | PD | SD | PR | CR | DCR | *p-*value |
| --- | --- | --- | --- | --- | --- | --- |
| Others group vs. Endocrine irAEs group |  |  |  |  |  | 0.006 |
| Others | 28 | 107 | 38 | 0 | 83.8% |  |
| Endocrine irAEs | 0 | 30 | 8 | 2 | 100% |  |
| Others group vs. Dermatologic irAEs group |  |  |  |  |  | 0.435 |
| Others | 28 | 132 | 41 | 2 | 86.2% |  |
| Dermatologic irAEs | 0 | 5 | 5 | 0 | 100.0% |  |
| Others group vs. Hepatic irAEs group |  |  |  |  |  | 1.000 |
| Others | 27 | 130 | 44 | 2 | 86.7% |  |
| Hepatic irAEs | 1 | 7 | 2 | 0 | 90.0% |  |
| Others group vs. Pulmonary irAEs group |  |  |  |  |  | 0.643 |
| Others | 27 | 127 | 41 | 2 | 86.3% |  |
| Pulmonary irAEs | 1 | 10 | 5 | 0 | 93.8% |  |

Other patients, including all the patients except the corresponding organ-specific irAE patients; IrAEs, immune-related adverse events; Vs, versus; SD, stable disease; PR, partial response; PD, progressive disease; CR, complete response; DCR, disease control rate.

Table S7. Median PFS and OS of each organ-specific group vs. other patients.

| Covariate | Median PFS (95% CI)  (months) | *p-*value  (Log-rank) | Median OS (95% CI)  (months) | *p-*value  (Log-rank) |
| --- | --- | --- | --- | --- |
| Endocrine irAEs |  | <.001 |  | 0.006 |
| Others | 10.67 (8.76 - 12.50) |  | 15.33 (13.05 - 17.62) |  |
| Endocrine irAEs | 18.33 (10.19 – 26.48) |  | 24.37 (15.37 – 33.36) |  |
| Dermatologic irAEs  Others  Dermatologic irAE | 12.47 (10.21 - 14.72)  11.70 (8.66 – 14.75) | 0.606 | 16.77 (13.65 - 19.89)  18.47 (2.61 – 34.32) | 0.487 |
| Hepatic irAEs  Others  Hepatic irAEs | 12.30 (10.16 - 14.44)  11.53 (3.42 – 19.64) | 0.150 | 16.77 (14.10 - 19.43)  27.77 (23.03 – 32.50) | 0.075 |
| Pulmonary irAEs  Others  Pulmonary irAEs | 12.30 (10.00 - 14.60)  10.83 (5.22 – 16.45) | 0.371 | 16.77 (13.99 - 19.55)  27.27 (5.57 – 48.97) | 0.082 |

Other patients, including all the patients except the corresponding organ-specific irAE patients; IrAEs, immune-related adverse events; Vs, versus; CI, confidence interval; OS, overall survival; PFS, progression free survival.

Table S8. Univariate Cox analysis of organ-specific irAEs and PFS in ESCC patients.

| Covariate | Univariate Analysis | |
| --- | --- | --- |
|  | HR (95% CI) | *p*-value |
| Endocrine irAEs |  |  |
| Others | 1.00 (Reference) |  |
| Endocrine irAEs | 0.45 (0.28 - 0.72) | <.001 |
| Dermatologic irAEs |  |  |
| Others | 1.00 (Reference) |  |
| Dermatologic irAEs | 0.83 (0.41 - 1.69) | 0.607 |
| Hepatic irAEs |  |  |
| Others | 1.00 (Reference) |  |
| Hepatic irAEs | 0.57 (0.27 - 1.23) | 0.155 |
| Pulmonary irAEs |  |  |
| Others | 1.00 (Reference) |  |
| Pulmonary irAEs | 0.77 (0.43 - 1.37) | 0.372 |

Other patients, including all the patients except the corresponding organ-specific irAE patients; IrAEs, immune-related adverse events; HR, hazard ratio; CI, confidence interval; PFS, progression free survival; ESCC, esophageal squamous cell carcinoma; PFS, progression free survival.

Table S9. Univariate Cox analysis of organ-specific irAEs and OS in ESCC patients.

| Covariate | Univariate Analysis | |
| --- | --- | --- |
|  | HR (95% CI) | *p*-value |
| Endocrine irAEs |  |  |
| Others | 1.00 (Reference) |  |
| Endocrine irAEs | 0.49 (0.29 - 0.82) | 0.007 |
| Dermatologic irAEs |  |  |
| Others | 1.00 (Reference) |  |
| Dermatologic irAEs | 0.76 (0.36 - 1.64) | 0.489 |
| Hepatic irAEs |  |  |
| Others | 1.00 (Reference) |  |
| Hepatic irAEs | 0.48 (0.21 - 1.10) | 0.082 |
| Pulmonary irAEs |  |  |
| Others | 1.00 (Reference) |  |
| Pulmonary irAEs | 0.55 (0.28 - 1.09) | 0.087 |

Other patients, including all the patients except the corresponding organ-specific irAE patients; IrAEs, immune-related adverse events; HR, hazard ratio; CI, confidence interval; OS, overall survival; ESCC, esophageal squamous cell carcinoma; OS, overall survival.

Table S10. Response to ICI of irAEs, non-irAE, mild-irAE, and severe-irAE groups in ESCC patients.

| Comparison | PD | SD | PR | CR | DCR | *p-*value |  |
| --- | --- | --- | --- | --- | --- | --- | --- |
| Non-irAE group vs Mild-irAE group |  |  |  |  |  | 0.012 |  |
| Non-irAE group | 25 | 81 | 28 | 0 | 82.1% |  |  |
| Mild-irAE group | 2 | 43 | 16 | 2 | 95.2% |  |  |
| Non-irAE group vs Severe-irAE group |  |  |  |  |  | 0.475 |  |
| Non-irAE group | 25 | 81 | 28 | 0 | 82.1% |  |  |
| Severe-irAE group | 1 | 13 | 2 | 0 | 93.8% |  |  |
| Mild-irAE group vs Severe-irAE group  Mild-irAE group  Severe-irAE group | 2  1 | 43  13 | 16  2 | 2  0 | 95.2%  93.8% | 1.000 |  |

IrAEs, immune-related adverse events; SD, stable disease; PR, partial response; PD, progressive disease; CR, complete response; DCR, disease control rate; ESCC, esophageal squamous cell carcinoma.

Table S11. Median PFS and OS of non-irAE, mild-irAE, and severe-irAE groups in ESCC patients.

| Covariate | Median PFS (95% CI)  (months) | *p-*value  (Log-rank) | Median OS (95% CI)  (months) | *p-*value  (Log-rank) |
| --- | --- | --- | --- | --- |
| Non-irAE group vs Mild-irAE group |  | ＜0.001 |  | ＜0.001 |
| Non-irAE group | 10.67 (7.59- 13.74) |  | 15.27 (12.87 – 17.66) |  |
| Mild-irAE group | 17.50 (11.15 - 23.85) |  | 22.00 (13.23 – 30.77) |  |
| Non-irAE group vs Severe-irAE group |  | 0.490 |  | 0.145 |
| Non-irAE group | 10.67 (7.59- 13.74) |  | 15.27 (12.87 – 17.66) |  |
| Severe -irAE group | 9.33 (7.47 - 11.13) |  | 17.67 (0.00 – 42.12) |  |
| Mild-irAE group vs Severe-irAE group |  | 0.144 |  | 0.407 |
| Mild-irAE group | 17.50 (11.15 - 23.85) |  | 22.00 (13.23 – 30.77) |  |
| Severe -irAE group | 9.33 (7.47 - 11.13) |  | 17.67 (0.00 – 42.12) |  |

IrAEs, immune-related adverse events; OS, overall survival; PFS, progression free survival; ESCC, esophageal squamous cell carcinoma.

.

Table S12. Univariate and Multivariate Cox Proportional Hazards Analysis of PFS by irAE severity.

| Variables | Univariate Analysis | | Multivariate Analysis | |
| --- | --- | --- | --- | --- |
|  | HR (95%CI) | *p-*value | HR (95%CI) | *p-*value |
| Gender |  |  |  |  |
| Female | 1.00 (Reference) |  |  |  |
| Male | 1.10 (0.78 - 1.54) | 0.595 |  |  |
| Age Group |  |  |  |  |
| <65 | 1.00 (Reference) |  |  |  |
| ≥65 | 0.93 (0.67 - 1.29) | 0.659 |  |  |
| ECOG |  |  |  |  |
| ≤1 | 1.00 (Reference) |  | 1.00 (Reference) |  |
| ＞1 | 2.23 (1.52 - 3.29) | <.001 | 2.10 (1.41 - 3.12) | <.001 |
| NLR |  |  |  |  |
| NLR＜3 | 1.00 (Reference) |  |  |  |
| NLR≥3 | 1.11 (0.81 - 1.52) | 0.533 |  |  |
| TNM stage |  |  |  |  |
| II | 1.00 (Reference) |  |  |  |
| III | 1.49 (0.83 - 2.66) | 0.182 |  |  |
| IV | 1.70 (0.97 - 3.00) | 0.065 |  |  |
| Tumor differentiation |  |  |  |  |
| Medium to high | 1.00 (Reference) |  |  |  |
| Low | 1.08 (0.72 - 1.62) | 0.721 |  |  |
| Tumor site |  |  |  |  |
| Upper | 1.00 (Reference) |  |  |  |
| Middle | 0.88 (0.57 - 1.36) | 0.561 |  |  |
| Lower | 0.84 (0.53 - 1.34) | 0.470 |  |  |
| Other | 0.74 (0.46 - 1.19) | 0.215 |  |  |
| PD-L1 expression |  |  |  |  |
| Negative | 1.00 (Reference) |  |  |  |
| Positive | 1.15 (0.63 - 2.10) | 0.638 |  |  |
| Unknown | 1.49 (0.87 - 2.56) | 0.150 |  |  |
| History of surgery |  |  |  |  |
| No | 1.00 (Reference) |  |  |  |
| Yes | 1.01 (0.57 - 1.78) | 0.976 |  |  |
| Number of metastases |  |  |  |  |
| ≤1 | 1.00 (Reference) |  | 1.00 (Reference) |  |
| ＞1 | 1.61 (1.15 - 2.25) | 0.006 | 1.63 (1.16 - 2.30) | 0.005 |
| Chemotherapy regimen |  |  |  |  |
| Others | 1.00 (Reference) |  | 1.00 (Reference) |  |
| TP | 0.54 (0.36 - 0.80) | 0.002 | 0.58 (0.39 - 0.87) | 0.009 |
| IrAEs groups |  |  |  |  |
| Non-irAE | 1.00 (Reference) |  | 1.00 (Reference) |  |
| Mild-irAE | 0.57 (0.39 - 0.82) | 0.003 | 0.53 (0.36 - 0.78) | 0.001 |
| Severe-irAE | 0.61 (0.34 - 1.10) | 0.100 | 0.77 (0.43 - 1.37) | 0.377 |

IrAEs, immune-related adverse events; ESCC, esophageal squamous cell carcinoma; HR, hazard ratio; NLR, Neutrophil to Lymphocyte Ratio; ECOG, Eastern Cooperative Oncology Group; TNM, tumor-node-metastasis; PD-L1, programmed cell death ligand 1; PFS, progression free survival.

Table S13. Univariate and Multivariate Cox Proportional Hazards Analysis of OS by irAE severity.

| Variables | Univariate Analysis | | Multivariate Analysis | |
| --- | --- | --- | --- | --- |
|  | HR (95%CI) | *p-*value | HR (95%CI) | *p-*value |
| Gender |  |  |  |  |
| Female | 1.00 (Reference) |  |  |  |
| Male | 1.18 (0.82 - 1.69) | 0.383 |  |  |
| Age Group |  |  |  |  |
| <65 | 1.00 (Reference) |  |  |  |
| ≥65 | 0.92 (0.65 - 1.29) | 0.623 |  |  |
| ECOG |  |  |  |  |
| ≤1 | 1.00 (Reference) |  | 1.00 (Reference) |  |
| ＞1 | 2.15 (1.43 - 3.24) | <.001 | 2.10 (1.39 - 3.18) | <.001 |
| NLR |  |  |  |  |
| NLR＜3 | 1.00 (Reference) |  |  |  |
| NLR≥3 | 1.29 (0.92 - 1.80) | 0.142 |  |  |
| TNM stage |  |  |  |  |
| II | 1.00 (Reference) |  |  |  |
| III | 1.00 (0.55 - 1.81) | 0.996 |  |  |
| IV | 1.27 (0.72 - 2.25) | 0.409 |  |  |
| Tumor differentiation |  |  |  |  |
| Medium to high | 1.00 (Reference) |  |  |  |
| Low | 0.79 (0.50 - 1.25) | 0.316 |  |  |
| Tumor site |  |  |  |  |
| Upper | 1.00 (Reference) |  |  |  |
| Middle | 1.00 (0.63 - 1.59) | 0.993 |  |  |
| Lower | 1.00 (0.61 - 1.64) | 0.989 |  |  |
| Other | 0.87 (0.53 - 1.43) | 0.575 |  |  |
| PD-L1 expression |  |  |  |  |
| Negative | 1.00 (Reference) |  |  |  |
| Positive | 0.97 (0.51 - 1.86) | 0.935 |  |  |
| Unknown | 1.34 (0.75 - 2.39) | 0.329 |  |  |
| History of surgery |  |  |  |  |
| No | 1.00 (Reference) |  |  |  |
| Yes | 1.02 (0.57 - 1.81) | 0.955 |  |  |
| Number of metastases |  |  |  |  |
| ≤1 | 1.00 (Reference) |  | 1.00 (Reference) |  |
| ＞1 | 1.75 (1.23 - 2.50) | 0.002 | 1.70 (1.19 - 2.44) | 0.004 |
| Chemotherapy regimen |  |  |  |  |
| Others | 1.00 (Reference) |  |  |  |
| TP | 0.68 (0.45 - 1.02) | 0.060 |  | 0.006 |
| IrAEs groups |  |  |  |  |
| Non-irAE | 1.00 (Reference) |  | 1.00 (Reference) |  |
| Mild-irAE | 0.50 (0.33 - 0.75) | <.001 | 0.54 (0.36 - 0.82) | 0.004 |
| Severe-irAE | 0.64 (0.35 - 1.16) | 0.006 | 0.70 (0.38 - 1.29) | 0.251 |

IrAEs, immune-related adverse events; ESCC, esophageal squamous cell carcinoma; HR, hazard ratio; NLR, Neutrophil to Lymphocyte Ratio; ECOG, Eastern Cooperative Oncology Group; TNM, tumor-node-metastasis; PD-L1, programmed cell death ligand 1; OS, overall survival.


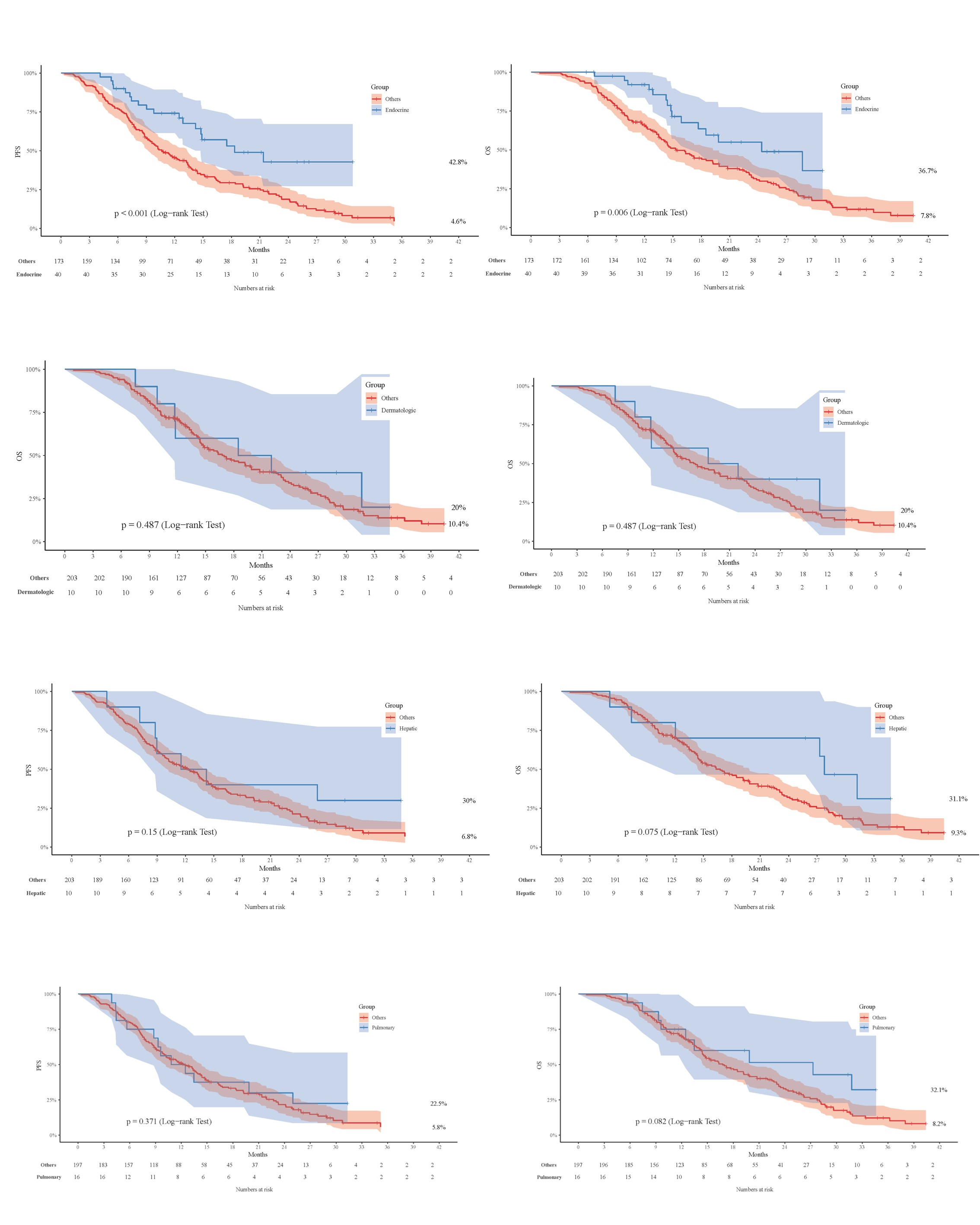


Figure S1. **(A.-J)** The Kaplan–Meier curve of progression-free survival (PFS) and overall survival (OS) of each specific irAE with a case number of more than 10 patients in ESCC patients. **(A)** PFS of endocrine irAE patients. **(B)** OS of endocrine irAE patients. **(C)** PFS of dermatologic irAE patients. **(D)** OS of dermatologic irAE patients. **(E)** PFS of hepatic irAE patients. **(F)** OS of hepatic irAE patients. **(G)** PFS of pulmonary irAE patients. **(H)** OS of pulmonary irAE patients.
